# Assessing the Contribution of Malaria Vector Control and Other Maternal and Child Health Interventions in Reducing All-Cause Under-Five Mortality in Zambia, 1990–2010

**DOI:** 10.4269/ajtmh.15-0315

**Published:** 2016-02-15

**Authors:** Marie Ng, K. Ellicott Colson, Nancy Fullman, Laura Dwyer-Lindgren, Tom Achoki, Matthew T. Schneider, Peter Mulenga, Peter Hangoma, Felix Masiye, Emmanuela Gakidou

**Affiliations:** 1 Institute for Health Metrics and Evaluation, University of Washington, Seattle, Washington;; 2 University of California, Berkeley, Berkeley, California;; 3 United States Agency for International Development, Washington, District of Columbia;; 4 Clinton Health Access Initiative, Lusaka, Zambia;; 5 Department of Economics, University of Bergen, Bergen, Norway;; 6 School of Humanities and Social Sciences, University of Zambia, Lusaka, Zambia

## Abstract

Under-five mortality in Zambia has declined since 1990, with reductions accelerating after 2000. Zambia’s scale-up of malaria control is viewed as the driver of these gains, but past studies have not fully accounted for other potential factors. This study sought to systematically evaluate the impact of malaria vector control on under-five mortality. Using a mixed-effects regression model, we quantified the relationship between malaria vector control, other priority health interventions, and socioeconomic indicators and district-level under-five mortality trends from 1990 to 2010. We then conducted counterfactual analyses to estimate under-five mortality in the absence of scaling up malaria vector control. Throughout Zambia, increased malaria vector control coverage coincided with scaling up three other interventions: the pentavalent vaccine, exclusive breast-feeding, and prevention of mother-to-child transmission of HIV services. This simultaneous scale-up made statistically isolating intervention-specific impact infeasible. Instead, in combination, these interventions jointly accelerated declines in under-five mortality by 11% between 2000 and 2010. Zambia’s scale-up of multiple interventions is notable, yet our findings highlight challenges in quantifying program-specific impact without better health data and information systems. As countries aim to further improve health outcomes, there is even greater need—and opportunity—to strengthen routine data systems and to develop more rigorous evaluation strategies.

## INTRODUCTION

Over the last two decades, substantial investments have been made to expand access to malaria interventions throughout sub-Saharan Africa. Development assistance for health (DAH) targeting malaria programs escalated from $88.6 million in U.S. dollars (USD) in 2003 to about $1.26 billion USD in 2010.^1^ During this time, many countries, including Zambia, also recorded marked declines in under-five mortality.^2^ As an early recipient of financial support from the President’s Malaria Initiative (PMI) and the Global Fund to Fight AIDS, Tuberculosis and Malaria,^3^^,^^4^ Zambia saw malaria DAH rise from $7.39 million USD in 2003 to $24.2 million USD in 2010.^1^

The scale-up of malaria vector control interventions, such as insecticide-treated nets (ITNs) and indoor residual spraying (IRS), has been viewed a primary driver of gains in child survival.^5^^–^^9^ However, minimal evidence exists on the specific impact of malaria vector control in reducing under-five mortality. To date, studies assessing the impact of malaria vector control interventions have not systematically accounted for secular changes in child health and trends in socioeconomic factors. Such work often cites descriptive findings as evidence to support the impact of malaria vector control, referring to declines in under-five mortality alongside increasing levels of ITN coverage and other malaria interventions.^8^^–^^11^ Yet many countries, including Zambia, also recorded improving socioeconomic conditions during this time,^12^ as well as greater access a range of key maternal and child health (MCH) interventions, including immunizations^13^ and prevention of mother-to-child transmission of HIV (PMTCT) services.^14^ In addition, Zambia was among one of the first countries that formally adopted artemisinin-based combination therapies (ACTs) as its first-line antimalarial in 2002, a policy decision that followed rising rates of treatment failure from chloroquine and escalating malaria-attributable mortality.^15^ Such gains have likely contributed to improved childhood survival as well,^16^^–^^18^ and failing to account for them, alongside the scale-up of vector-focused malaria interventions, may lead to misleading results from evaluation studies.

Systematically evaluating the impact of malaria vector control interventions involves overcoming a number of methodological challenges. Program implementation is rarely uniform across geographies,^8^ and interventions are frequently delivered in a phased manner over time.^19^^,^^20^ Areas with higher malaria transmission are typically targeted to receive more malaria control interventions; if these high burden areas also have higher rates of under-five mortality, it may appear that receiving malaria interventions is related to heightened death rates rather than a result of successful program targeting.^21^^,^^22^ Although capturing local variations in disease burden and program inputs is of high analytic priority,^23^ reliably collected data tracking subnational program activities and health outcomes over time are generally sparse. These data limitations can substantially hinder tracking local progress in evidence-driven ways.^17^^,^^24^

With this study, we sought to quantify the specific contribution of malaria vector control interventions in Zambia’s reductions in under-five mortality, while accounting for other key MCH interventions and socioeconomic factors. To achieve this aim, we used district-level trends for a full range of indicators and developed a model to statistically examine the relationship between coverage of malaria vector control interventions and all-cause under-five mortality. We then conducted a counterfactual analysis, through which we could predict trends in under-five mortality in the absence of scaling up malaria vector control interventions.

## METHODS

Our analysis involved four main steps. First, we collated all available data on malaria interventions, key MCH interventions, socioeconomic factors, and under-five mortality in Zambia. Second, we generated district-level estimates, for each indicator and Zambia’s 72 districts, from 1990 to 2010. Third, we performed a series of mixed-effect regressions to quantify the relationship between coverage of malaria vector control interventions and under-five mortality. Fourth, we conducted counterfactual analyses to determine how much reductions in under-five mortality were attributable to the scale-up of malaria vector control and how much these declines were attributable to other health programs and socioeconomic gains.

### Data.

We used district-level time series generated from previous studies.^25^^,^^26^ For under-five mortality, district-level data were extracted from household surveys and population censuses, and then small area estimation techniques were applied to produce trend estimates for each of Zambia’s 72 districts.^25^ Briefly, small area estimation techniques take advantage of geographical relationships to derive estimates for a small geographical unit where direct estimates are not always available. For all health interventions and socioeconomic indicators, district-level time series were produced for each indicator through a space-time model and Gaussian process regression.^26^ Multiple sources of data, including a number of different household surveys and administrative databases, were used for this analysis. All data sources can be found in Supplemental Table 1, and additional information about estimation methods are detailed elsewhere.^25^^,^^26^

Table 1 summarizes the indicators included in our final analysis. One of the primary indicators of interest was coverage of malaria vector control interventions, which was represented by the proportion of households in a district that owned at least one ITN or had received IRS in the last 12 months, or had both ITNs and IRS. Additional malaria interventions, such as access to or the receipt of ACTs, were excluded due to challenges with data availability and quality. Key MCH indicators included coverage of measles immunization, the pentavalent vaccine, and the diphtheria–pertussis–tetanus vaccine, three doses (DPT3); exclusive breast-feeding; antenatal care, one visit (ANC1); the proportion of children who were not underweight; and the availability of PMTCT services. For PMTCT, we only had provincial-level data, so we use the same estimates for all districts within each province.

**Table 1:** Definitions of indicators used in the analysis

Indicator	Definition
Malaria vector control interventions	
ITN ownership or IRS	The proportion of households that owned at least one insecticide-treated net (ITN) or that were sprayed with an insecticide-based solution in the last 12 months (indoor residual spraying)
Immunizations	
DPT3 immunization	The proportion of children aged 12–59 months who received three doses of the diphtheria-pertussis-tetanus (DPT) vaccine
Measles immunization	The proportion of children aged 12–19 months who received measles vaccination
Pentavalent immunization	The proportion of children aged 12–59 months who received the pentavalent vaccine, which includes protection against DPT, hepatitis B, and *Haemophilus influenzae* type b
Other key maternal and child health interventions	
Antenatal care, one visit	The proportion of women aged 15–49 years who had one or more antenatal visits at a health facility during pregnancy
Exclusive breast-feeding	The proportion of children who were exclusively breast-fed during their first 6 months after birth
Childhood underweight	The proportion of children under five who were two or more standard deviations below the international anthropometric reference population median of weight for age
HIV/AIDS	
Prevention of mother-to-child transmission of HIV (PMTCT)	The number of district health facilities in a province offering services for the prevention of mother-to-child transmission of HIV among HIV-positive pregnant women, per population under age 1 year in the province
Socioeconomic factors	
Adult educational attainment	Average years of education for people aged 18 years and older
Improved sanitation	The proportion of households with access to improved sanitation facilities (a flush toilet or covered pit latrine)
Improved cooking fuel	The proportion of households that use an improved source of cooking fuel (e.g., kerosene, biogas)
Electricity	The proportion of households with electricity

HIV/AIDS = human immunodeficiency virus/acquired immune deficiency syndrome.

We also included the following socioeconomic indicators: educational attainment among adults aged 18 and older; the availability of improved sanitation and electricity in households; and household use of improved cooking fuel. Supplemental Table 2 summarizes variables that were initially considered for inclusion but ultimately were not used for our analyses.

### Statistical analysis.

#### Model development.

A series of models were used to systematically assess the relationship between the coverage of malaria vector control interventions and under-five mortality while accounting for a range of other factors. Here, we provide an analysis summary; more details are available in the Supplemental Appendix.

Our first step was to run a simple regression to verify the bivariate association between coverage of malaria vector control interventions and under-five mortality at the district levels. We then applied a mixed-effects model to quantify this relationship while accounting for a range of MCH interventions and socioeconomic indicators. This initial model provided a reasonable fit, but strong multicollinearity was found for trends in malaria vector control intervention coverage and a subset of MCH indicators (pentavalent vaccine coverage, exclusive breast-feeding, and availability of PMTCT services). Since the scale-up of these interventions occurred concurrently, we used principal component analysis (PCA) to combine them into a single indicator reflecting rapidly scaled up interventions.

Our final model was as follows:

$$\begin{array}{c} \ln{\left(5q0\right)}_{i,k,t}=\beta_{0}+\beta_{1}{\mathrm{Scaled}}_{i,t}+\beta_{2}{SES}_{i,t}+\beta_{3}\left(1-{Und}_{i,t}\right)+\;\beta_{4}{\mathrm{Scaled}}_{i,t}\times {SES}_{i,t}+\beta_{5}\left(1-{Und}_{i,t}\right)\\ \times {SES}_{i,t}+\beta_{6}ANC1_{i,t}+\beta_{7}DPT3_{i,t}+\;\beta_{8}{\mathrm{Meas}}_{i,t}+\beta_{9}t+\mu_{k}+\epsilon_{i,k,t} \end{array}$$

where ln(5q0) was the log of the under-five mortality rate in district *i*, province *k*, and year *t*; Scaled_*i,t*_ was the composite variable derived through PCA, combining the coverage of ITNs or IRS with three other rapidly scaled up MCH interventions (pentavalent vaccine, exclusive breast-feeding, and availability of PMTCT services per capita under 1 year) in district *i* and year *t*; SES_*i,t*_ was a composite socioeconomic indicator, a PCA-generated weighted average of the mean years of education for individuals aged 18 and older, household availability of improved sanitation, household use of improved cooking fuel, and access to household electricity in district *i* and year *t*; Und_*i,t*_ was the proportion of children under five who were underweight in district *i* and year t; ANC1_*i,t*_ was coverage of ANC1 in district *i* and year *t*; DPT3_*i,t*_ was DPT3 immunization coverage in district *i* and year *t*; Meas_*i,t*_ was measles immunization coverage in district *i* and year *t*; *t* was a time variable intended to account for secular trends in under-five mortality; μ_*k*_ was a random intercept intended to capture heterogeneity in levels of under-five mortality across provinces; and ϵ_*i,k,t*_ was the error term. Two interaction terms, Scaled_*i,t*_ × SES_*i,t*_ and (1 − Und_*i,t*_) × SES_*i,t*_ were included in the model to capture the differential relationship between changes in under-five mortality and rapidly scaled up interventions, as well as the prevalence of childhood underweight across socioeconomic levels. *P*-values and 95% confidence intervals (CIs) for all coefficients were derived using bootstrapping. Further details are provided in the Supplemental Appendix.

A series of diagnostics were conducted to test analytic rigor and model fit. Each model’s performance in capturing spatial and temporal trends in the data was assessed by residual plots and goodness-of-fit tests. Cross-validation analyses were performed to address the potential risk of model overfitting. Finally, sensitivity analyses were conducted to test model robustness against data perturbation.

### Counterfactual analysis.

Using results from the previous model, we conducted counterfactual analyses to predict: 1) expected under-five mortality rates in 2010 and 2) changes in under-five mortality between 2000 and 2010 if coverage of the rapidly scaled up interventions had remained at 2000 and then 2005 levels of coverage. By comparing observed trends in under-five mortality with counterfactual values, we then estimated how much the scale-up of these interventions contributed to reductions in under-five mortality.

All statistical analyses were conducted with R (version 3.0.1; R Foundation for Statistical Computing, Vienna, Austria).

## RESULTS

**Figure 1.**
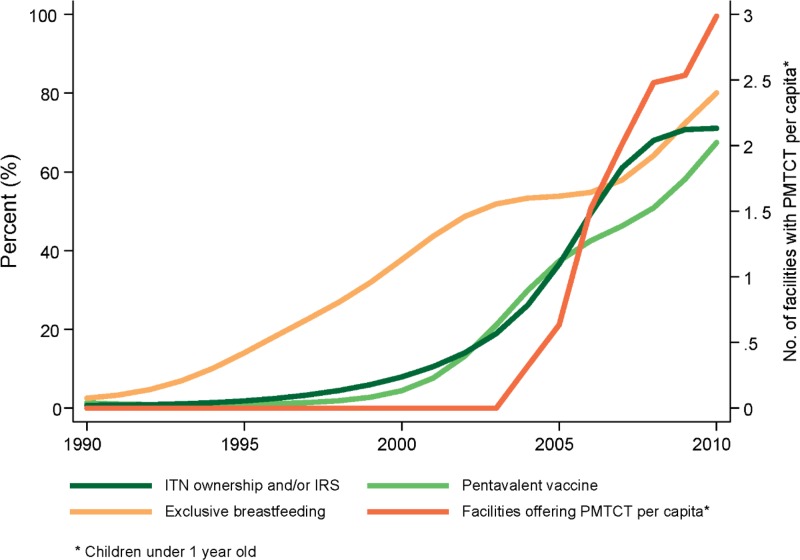
Simultaneous scale-up of multiple key maternal and child health interventions in Zambia, 1990–2010.

Figure 1 illustrates the extent to which Zambia’s coverage of malaria vector control rapidly increased alongside a subset of MCH interventions (pentavalent vaccine coverage, exclusive breast-feeding, and the availability of PMTCT services). Correlations between the malaria vector control indicator and these MCH interventions ranged from 0.77 to 0.90 (Supplemental Table 3). Such strong correlations across interventions made isolating the impact of malaria vector control from the impact of these rapidly scaled up MCH interventions analytically infeasible.

By using the PCA-generated composite indicator for these rapidly scaled up interventions, our mixed-effects regression model showed that increasing coverage of these interventions, in combination, were significantly associated with declines in under-five mortality (β_1_ = −0.024 [95% CI = −0.034, −0.013]) (Table 2). Coverage of DPT3 immunization was also significantly associated with reductions in under-five mortality (β_7_ = −0.184 [95% CI = −0.273, −0.109]). Diagnostic tests revealed that the final model performed well in accounting for geographical and serial correlation. Moreover, compared with the bivariate regression model, models that accounted for other MCH interventions and socioeconomic indicators demonstrated superior performance for both in-sample fit and predictive validity (Supplemental Appendix).

**Table 2:** Mixed-effects regression results

Indicator	Model 3		
	Coefficient	Standard error	95% Confidence intervals
Intercept	35.712*	3.063	41.715, 30.154
Composite of rapidly scaled up interventions (including: ITN ownership or IRS, PMTCT, exclusive breast-feeding, and pentavalent immunization)	−0.024*	0.005	−0.034, −0.013
SES	−0.117	0.082	−0.287, 0.044
Not underweight	−0.257	0.142	−0.539, 0.020
Composite of rapidly scaled up interventions: SES	0.003	0.002	−0.00005, 0.007
Not underweight: SES	0.110	0.099	−0.077, 0.305
ANC1	−0.123	0.067	−0.240, 0.007
DPT3 immunization	−0.184*	0.038	−0.273, −0.109
Measles immunization	0.155	0.080	−0.001, 0.311
Year	−0.015*	0.001	−0.018, −0.012
Overall goodness of fit (measured by CV RMSE)	0.0839		

ANC1 = antenatal care, one visit; CV RMSE = cross-validated root mean squared error; DPT3 = diphtheria-pertussis-tetanus vaccine, three doses; IRS = indoor residual spraying; ITN = insecticide-treated net; PMTCT = prevention of mother-to-child transmission of HIV; SES = socioeconomic status.

* *P* < 0.05.

**Figure 2.**
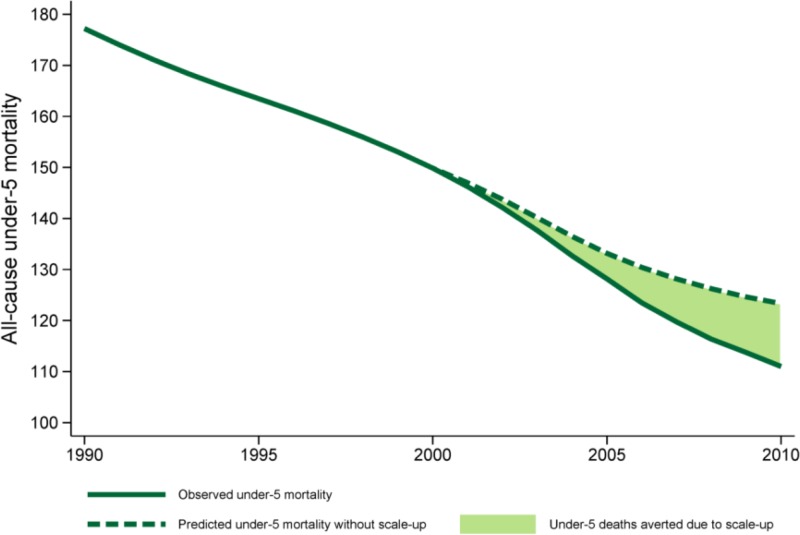
Trends in under-five mortality in Zambia as observed and predicted under the counterfactual of 2000 coverage levels for rapidly scaled up interventions. This figure appears in color at www.ajtmh.org.

Our counterfactual analyses indicated that, if coverage of malaria vector control interventions and the three other rapidly scaled up MCH interventions remained at 2000 levels, under-five mortality in Zambia would have been 124 deaths per 1,000 live births (95% CI = 118, 129) in 2010—11% higher than what was estimated for 2010 (111 deaths per 1,000 live births [95% CI = 108, 115]). Figure 2 depicts trends in under-five mortality under this counterfactual scenario. If coverage remained at 2005 levels from 2005 to 2010, we predicted that Zambia’s under-five mortality would be 119 deaths per 1,000 live births (95% CI = 118, 129), or 7% higher than the country’s observed mortality rate.

In comparing counterfactual and observed rates of annualized percent change in under-five mortality from 2000 to 2010, we found that Zambia’s declines in under-five mortality would not have been as rapid without the country’s marked scale-up of a subset of interventions (Table 3). Specifically, if coverage of the rapidly scaled up interventions remained at 2000 levels, Zambia’s rates of under-five mortality would have declined at an average of 1.7% per year (95% CI = 1.5%, 1.9%). If coverage remained at 2005 levels, annualized rates of reduction would have been 1.9% per year (95% CI = 1.7%, 2.1%), a rate moderately slower than the observed rate of decline from 2000 to 2010 (an average of 2.2% reduction in under-five mortality per year [95% CI = 2.0%, 2.4%]).

**Table 3:** Counterfactual under-five mortality rates

Scenario	Deaths per 1,000 live births in 2010 (95% CI)	Annualized rate of decline, 1990–2010 (95% CI)
Currently estimated values	111 (108, 115)	2.2% (2.0–2.4%)
Counterfactual at 2005 coverage levels	119 (114, 123)	1.9% (1.7–2.1%)
Counterfactual at 2000 coverage levels	124 (118, 129)	1.7% (1.5–1.9%)

CI = confidence interval.

In combination, these results indicate that Zambia’s rapid scale-up of a subset of MCH interventions, including malaria vector control, accelerated the country’s declines in under-five mortality, particularly between 2005 and 2010.

## DISCUSSION

This study represents the first-ever impact evaluation, in Zambia or for other countries in sub-Saharan Africa, wherein the effects of malaria vector control interventions on reducing under-five mortality were assessed alongside a range of other drivers that aim to improve child survival. By harnessing two decades of district-level trend data, we found that Zambia’s declines in under-five mortality were likely due to the simultaneous scale-up of multiple MCH interventions, including malaria vector control.

Our study underscores the importance of accounting for contextual factors when conducting impact evaluations.^27^ On their own, malaria vector control interventions were significantly related to declines in under-five mortality, an association consistently found by previous studies.^9^ The strength of this relationship diminished when we accounted for trends in other MCH interventions and socioeconomic indicators. This result shows that the scale-up of malaria vector control interventions was not the sole driver of reductions in Zambia’s under-five mortality. At the same time, statistically isolating the specific contribution of malaria vector control, as compared with other rapidly scaled up interventions, was analytically infeasible due to the nature of their concurrent scale-up of coverage.

For many evaluation studies, systematically including contextual factors has been hindered by data availability. Such work often relies on limited types of data sources, such as cross-sectional surveys or administrative databases. By drawing from only one or two types of data, analyses are frequently limited in scope.^28^ In using a broad range of data types to generate a full time series of district-level trends for under-five mortality, a range of MCH interventions, and socioeconomic indicators, our analysis could better capture geographical and temporal variations, more systematically account for secular trends, and detect intervention effects with greater precision.

As more countries aim to further reduce and ultimately eliminate malaria, there is an imminent need for systematically evaluating the effectiveness of intervention packages and strategies.^29^ To improve the scientific rigor of future impact evaluations, several aspects deserve further consideration. First, routine health information systems, particularly those at subnational levels, need to be substantially strengthened. Such efforts would not only include consolidating currently available health information into a centralized, consistent platform, but also expanding the representativeness of routinely administered surveys to subnational levels. Second, a more systematic and structured framework is necessary to guide future impact evaluation work. The PMI impact evaluation framework has highlighted essential features of high-equality evaluations,^30^ and the Roll Back Malaria Partnership has recently revised its evaluation framework for malaria programs,^31^ including the promotion of subnational analyses. Third, strategically planning—and prioritizing—prospective evaluations prior to and during program implementation is critical. Although it is frequently infeasible and ethnically inappropriate to roll out health programs using a randomized-control trial, a number of alternative evaluation strategies offer the opportunity to better establish the causal relationship between intervention scale-up and its impact on health outcomes. A stepped-wedge design, for instance, takes advantage of the phased implementation that is often logistically necessary to rolling out health interventions or programs.^32^^,^^33^

### Limitations.

Despite its methodological strengths, our study was subject to limitations. First, using ITN ownership as a main malaria vector control indicator may be a less direct measure of impact on child health outcomes than ITN use by children under five. Several household surveys were administered during the dry season (a time of lower risk for malaria transmission), and thus, reports of ITN use for the previous night would not necessarily reflect optimal intervention use. No rigorously tested correction factor is currently available to crosswalk between measures of ITN use during the dry and rainy seasons, so we opted to use household ownership of ITNs as a more stable indicator for malaria vector control. Second, some malaria indicators, such as malaria case management or ACT use, and MCH interventions that can benefit child health outcomes were not included in the current analysis due to data scarcity or substantive measurement issues. For example, household survey questions provide an imprecise indicator of ACT use, the proportion of children who received ACTs in response to having a fever in the last 2 weeks; greater measurement issues and challenges with small sample sizes then occurred when household surveys were administered during the dry season and we sought to analyze district-level ACT coverage. Further, district-level administrative data on ACTs were limited to monthly or annual drug procurement records and could not be linked to the number of children under five who were diagnosed with malaria and thus needed ACTs (the denominator for estimating intervention coverage), and those who then received full regimens of ACTs (the numerator). Third, we could not account for various factors that can affect intervention effectiveness, including measures of optimal intervention use and adherence, insecticide resistance, and intervention quality.^34^^–^^37^ Fourth, we did not analyze the potential for differential effects of malaria vector control across transmission settings, such that the impact of ITN and/or IRS may vary by the intensity of an area’s malaria burden.^38^ However, we tested the interaction between coverage of malaria vector control interventions and malaria transmission intensity, as measured by levels of *Pf*PR_2–10_,^39^ and did not differ markedly (see Supplemental Table 8 for details). Fifth, all estimates of under-five mortality and intervention coverage were derived from small area estimation methods and data synthesis techniques. Their corresponding levels of uncertainty could be not fully propagated throughout the current analysis, which theoretically may have resulted in underestimating the uncertainty of intervention impact. However, it is unlikely that this estimation limitation substantially affected our results or interpretation given the high levels of statistical significance between under-five mortality and the composite metric for rapidly scaled up intervention coverage (*P* < 0.0001).

## CONCLUSION

Like many countries in sub-Saharan Africa, Zambia has achieved marked success in reducing under-five mortality. The country’s scale-up of several MCH interventions, including malaria vector control, contributed to improved child survival, but due to their simultaneous increases in intervention coverage, the specific impact of malaria vector control could not be isolated from gains recorded for other MCH interventions. The analytical challenges and limitations highlighted by this study are not inherent obstacles to all impact evaluations; instead, with strengthened routine data systems and prospective evaluation planning, we can bolster the evidence base for intervention impact and provide guidance on data-driven strategies to achieve zero malaria.

## Supplementary Material

Supplemental Table.
