# Supplementary material for: Assessing the Contribution of Malaria Vector Control and Other Maternal and Child Health Interventions in Reducing All-Cause Under-Five Mortality in Zambia, 1990–2010
Source: Am J Trop Med Hyg. 2016 Feb 15;97(3 Suppl):58–64. doi: 10.4269/ajtmh.15-0315 (PMC5619928; doi:10.4269/ajtmh.15-0315)
Supplement: Supplementary file 1 [file tpmd150315.SD1.pdf]

The following are supplemental materials and will be published online only

doi:10.4269/ajtmh.15-0315.s001

SUPPLEMENTAL APPENDIX

Section I. Data.

Supplemental Table 1
Data sources used in analysis

Data source	Years represented
Surveys	
Demographic and Health Survey	1992, 1996–1997, 2001–2002, 2007
Malaria Indicator Survey	2006, 2008, 2010, 2012
Multiple Indicator Cluster Survey	1999
Living Conditions Monitoring Survey	1996, 1998, 2002–2003, 2004–2005, 2006, 2010
Japan International Cooperation Agency Health Facility Census	2005–2006
Sexual Behavior Survey	2005, 2009
Household Health Coverage Survey	2008
Netmark Survey reports	2000, 2004
Population censuses	
National census	1990, 2000, 2010
Administrative sources	
Health Management Information System	2000–2008, 2009
National Malaria Control Center malaria intervention databases	2005–2010
National AIDS Council quarterly status reports of facility-level PMTCT services	2005–2009
Central Statistical Office HIV/AIDS projections	1985–2010
Medical Stores Limited drug supply and delivery records	2007–2011
Global Precipitation Climatology Center precipitation data	1986–2012
Malaria Atlas Project malaria endemicity (*Pf*PR_{2-10})	2010

HIV/AIDS = human immunodeficiency virus/acquired immune deficiency syndrome; PMTCT = prevention of mother-to-child transmission of HIV.

Supplemental Table 2
Indicators considered but excluded in the analysis

Indicator	Reasons for exclusion
Malaria	
ACT coverage	Too few cases per survey-district
IPTp2 coverage	Collinear with ITN ownership/IRS; insufficient data across districts and over time
Transmission intensity	No observed relationship with under-five mortality; only available for 2010
Rainfall	No observed relationship with under-five mortality
Immunizations	
Polio immunization	Not a major cause of under-five mortality in Zambia
BCG immunization	No observed relationship with under-five mortality
Other maternal and child health interventions	
Antenatal care, —two to four visits	Collinear with ANC1
Skilled birth attendance	No observed relationship with under-five mortality
Stunting	Does not capture acute malnutrition
Wasting	Does not capture chronic malnutrition
Nutritional interventions	Insufficient data across districts and over time
Vitamin A supplementation	Evidence of causal relationship with under-five mortality is weak
Diarrhea treatment	Too few cases per survey district
Pneumonia treatment	Too few cases per survey district
Prevalence of low birth weight	No observed relationship with under-five mortality
Measles campaigns and child health weeks	No corresponding observed mortality shocks
HIV/AIDS	
PMTCT coverage	Data not available
Pediatric ART	Data not available
HIV prevalence	District-level data quality issues; provincial data only available for 2001–2002 and 2007
Number of HIV-positive women on ART	Lack of appropriate denominator data
Socio-demographic factors	
Maternal education	Covariate used to model intervention coverage
Urbanicity	Collinear with electricity
Fertility	No observed relationship with under-five mortality
Birth spacing	No observed relationship with under-five mortality
Others	
HMIS under-five cases and deaths by cause	No data acquired for period 1990–1999; substantial missingness; documented data quality issues
Health facilities per capita	Covariate used to model intervention coverage
MSL drug distribution database	Data only available for 2007–2011; substantial missingness; lack of accurate denominator data
District health office expenditures	Insufficient data across districts and over time
Access to health facilities	Insufficient data across districts and over time

ACT = artemisinin-based combination therapy; ANC1 = antenatal care, one visit; ART = antiretroviral therapy; BCG = Bacille Calmette–Guerin; HIV/AIDS = human immunodeficiency virus/acquired immune deficiency syndrome; HMIS = Health Management Information System; IPTp2 = intermittent preventive treatment in pregnancy, two doses; IRS = indoor residual spraying; ITN = insecticide-treated net; MSL = Medical Stores Limited; PMTCT = prevention of mother-to-child transmission of HIV.

The following are supplemental materials and will be published online only

Supplemental Table 3
Correlation matrix of covariates

Indicator	SES	ITN/IRS	EBF	Pent	PMTCT	ANC1	DPT3	Measles	Not underweight	ln(5q0)
SES	1.00	0.16	0.17	0.13	0.15	0.28	0.17	0.25	0.41	–0.44
ITN/IRS		1.00	0.77	0.90	0.85	–0.02	0.32	0.36	0.41	–0.64
EBF			1.00	0.78	0.66	–0.01	0.16	0.36	0.46	–0.66
Pent				1.00	0.83	0.00	0.40	0.39	0.44	–0.64
PMTCT					1.00	–0.01	0.31	0.33	0.39	–0.61
ANC1						1.00	0.25	0.35	0.20	–0.22
DPT3							1.00	0.57	0.29	–0.36
Measles								1.00	0.35	–0.46
Not underweight									1.00	–0.65
ln(5q0)										1.00

ANC1 = antenatal care, one visit; DPT3 = diphtheria–pertussis–tetanus vaccine, three doses; EBF = exclusive breast-feeding; ITN/IRS = insecticide-treated net/indoor residual spraying; PMTCT = prevention of mother-to-child transmission of HIV; SES = socioeconomic status.

Section II. Model development. To systematically quantify the impact of malaria vector control interventions on under-5 mortality, a series of models were applied. First, to validate our data against previous findings, a simple regression model was used to explore the bivariate association between under-5 mortality and malaria vector control interventions. The model specification is as follows (Model 1):

$$\ln(5q0)_{i,k,t} = \beta_0 + \beta_1 ITNownIRS_{i,t} + \varepsilon_{i,k,t} \quad (1)$$

where $\ln(5q0)_{i,k,t}$ was the natural logarithm of under-five mortality in district i, province k, and year t; $\mathrm{ITNownIRS}_{i,t}$ was the percent of households with ITN ownership or IRS in district i and year t; and $\varepsilon_{i,k,t}$ was the error term. β_1 reflects the statistical association between the outcome and intervention coverage. However, this statistical association could be driven by confounding factors. To understand the unique contribution of malaria vector control interventions, a second model was fitted taking into account various other factors, such as coverage of other MCH interventions and socioeconomic indicators, which also can affect under-five mortality. Specifically, the following mixed-effects model was applied (Model 2):

$$\begin{aligned}\ln(5q0)_{i,k,t} = {} & \beta_0 + \beta_1 \mathrm{ITNownIRS}_{i,t} + \beta_2 \mathrm{SES} + \beta_3(1-\mathrm{Und}_{i,t}) \\ & + \beta_4 \mathrm{ITNownIRS}_{i,t} \times \mathrm{SES}_{i,t} + \beta_5(1-\mathrm{Und}_{i,t}) \\ & \times \mathrm{SES}_{i,t} + \beta_6 \mathrm{Pent}_{i,t} + \beta_7 \mathrm{EBF}_{i,t} + \beta_8 \mathrm{PMTCT}_{k,t} \\ & + \beta_9 \mathrm{ANC1}_{i,t} + \beta_{10} \mathrm{DPT3}_{i,t} + \beta_{11} \mathrm{Meas}_{i,t} + \beta_{12} t \\ & + \mu_k + \varepsilon_{i,k,t}\end{aligned} \quad (2)$$

where $\mathrm{SES}_{i,t}$ was a composite indicator representing the socioeconomic status in district i and year t. This indicator, generated through PCA, combined a set of socioeconomic variables including mean years of education among adults aged 18 and older, coverage of improved sanitation, coverage of improved cooking fuel, and household electricity availability. Other indicators included in the model were: $\mathrm{Und}_{i,t}$, the proportion of children underweight in district i and year t; $\mathrm{Pent}_{i,t}$, coverage of the pentavalent vaccine in district i and year t; $\mathrm{EBF}_{i,t}$, the proportion of children who were exclusively breast-fed for the first 6 months of life in district i and year t; $\mathrm{PMTCT}_{k,t}$, the number of district health facilities that offered PMTCT services per capita under 1 year in province k and year t; $\mathrm{ANC1}_{i,t}$, antenatal care coverage (one visit) in district i and year t; $\mathrm{DPT3}_{i,t}$, DPT immunization coverage (three doses) in district i and year t; $\mathrm{Meas}_{i,t}$, measles immunization (one dose) in district i and year t; t, a time variable included to account for secular trends in under-five mortality; μ_k a random intercept included to capture heterogeneity in the levels of under-five mortality across provinces, and $\varepsilon_{i,k,t}$ was the error term. Two interaction terms, $\mathrm{ITNownIRS}_{i,t} \times \mathrm{SES}_{i,t}$ and $(1-\mathrm{Und}_{i,t}) \times \mathrm{SES}_{i,t}$ were included in the model to capture the differential relationships between under-five mortality with malaria vector control interventions as well as underweight across socioeconomic status in district i and year t.

An analytical concern pertaining to Model 2 was the presence of multicollinearity. Because malaria vector control interventions and some MCH interventions have been concurrently scaled up between 2000 and 2010, a subset of indicators, including $\mathrm{ITNownIRS}_{i,t}$, $\mathrm{Pent}_{i,t}$, $\mathrm{EBF}_{i,t}$, and $\mathrm{PMTCT}_{k,t}$ were highly correlated. The presence of high correlation can potentially hamper a model's capacity to detect significant effects. To address this issue, we used a third model (i.e., final model presented in the main text), which considered a subset of rapidly scaled up interventions as a collective package. The specification of the model is as follows (Model 3):

$$\begin{aligned}\ln(5q0)_{i,k,t} = {} & \beta_0 + \beta_1 \mathrm{Scaled}_{i,t} + \beta_2 \mathrm{SES}_{i,t} + \beta_3 \mathrm{Und}_{i,t} \\ & + \beta_4 \mathrm{Scaled}_{i,t} \times \mathrm{SES}_{i,t} + \beta_5 \mathrm{Und}_{i,t} \times \mathrm{SES}_{i,t} \\ & + \beta_6 \mathrm{ANC1}_{i,t} + \beta_7 \mathrm{DPT3}_{i,t} + \beta_8 \mathrm{Meas}_{i,t} \\ & + \beta_9 t + \mu_k + \varepsilon_{i,k,t}\end{aligned} \quad (3)$$

where $\mathrm{Scaled}_{i,t}$ was a composite variable which combined the four rapidly scaled up interventions using PCA (i.e., malaria vector control interventions, as defined by household coverage of ITNs or IRS, and three other MCH interventions: the pentavalent vaccine, exclusive breast-feeding, and the availability of PMTCT services). Comparing the results of Models 2 and 3 allowed us to determine the legitimacy of attributing impact uniquely to malaria vector control interventions and recognizing the need for assessing these subsets of interventions in a holistic manner.

Coefficients of the various models are shown in Supplemental Table 4. In Model 1, a significant bivariate association was found between household ITN ownership or IRS and all-cause under-five mortality (β_1 = –0.510 [95% CI = –0.552, –0.468]). This significant relationship, however, no longer held in Model 2. After accounting for an indicator for socioeconomic status and other MCH interventions, the association between under-five mortality and household ITN ownership or IRS was weakened (β_1 = –0.050 [95% CI = –0.146, 0.057]).

Other interventions, including PMTCT availability, exclusive breast-feeding, and DPT3 were significantly related to under-five mortality. Specifically, increases in the coverage of these interventions were associated with decreases in under-five mortality. Although Models 2 and 3 both presented a comparable fit, the presence of multicollinearity undermined the validity of statistical inference; hence, Model 3 was preferred.

Section III. Bootstrap method for statistical inference. Given the presence of spatial and serial correlation in the data, classic *t*-statistics for testing coefficient significance was not considered analytically appropriate. To ensure the accuracy of statistical inference, we applied a block bootstrap approach. In particular, from the original dataset, we treated each district as a block and sampled with 72 replacement blocks to construct bootstrapped samples. We ran the respective models, and obtained bootstrapped estimates of coefficients. We repeated this process 599 times to derive bootstrapped betas, *P* values, and 95% CIs.[1]

Section IV. Model validation. We used various strategies to ascertain model accuracy. First, residuals plots and within-sample root mean squared errors (RMSEs) were generated to assess overall in-sample model fit. Second, cross-validation was used to evaluate the risk of overfitting. Finally, sensitivity analyses were conducted by fitting the final model on different subsets of data to determine the robustness of model specification.

Supplemental Table 4
Regression coefficients for each model

Indicator	Model 1 Beta (SE)	Model 2 Beta (SE)	Model 3 Beta (SE)
Intercept	5.092 (0.020)*	36.300 (3.806)*	35.712 (3.063)*
ITN ownership or IRS	−0.510 (0.021)*	−0.050 (0.054)	
PMTCT		−21.097 (6.610)*	
Exclusive breast-feeding		−0.069 (0.033)*	
Pentavalent immunization		0.039 (0.042)	
Not underweight		−0.249 (0.147)	−0.257 (0.142)
ANC1		−0.120 (0.063)	−0.123 (0.067)
DPT3 immunization		−0.217 (0.047)*	−0.184 (0.038)*
Measles immunization		0.158 (0.083)	0.155 (0.080)
SES		−0.121 (0.095)	−0.117 (0.082)
Year		−0.015 (0.002)*	−0.015 (0.001)*
ITN ownership or IRS: SES		0.017 (0.011)	
Not underweight: SES		0.110 (0.098)	0.110 (0.099)
Composite of rapidly scaled up interventions			−0.024 (0.005)*
Composite of rapidly scaled up interventions: SES			0.003 (0.002)

ANC1 = antenatal care, one visit; DPT3 = diphtheria-pertussis-tetanus vaccine, three doses; IRS = indoor residual spraying; ITN = insecticide-treated net; PMTCT = prevention of mother-to-child transmission of HIV; SE = standard error; SES = socioeconomic status.
*$P < 0.05$.

Model diagnostics. RMSEs showed that Models 2 and 3 offered similar results and were considerably better at accounting for data variability as compared with the simple regression model (Model 1; Supplementary Table 5). Model 1's residual plot exhibited distinct patterns, indicating that some relationships between the data had not been adequately captured. By contrast, residual plots for Models 2 and 3 appeared to be more

The following are supplemental materials and will be published online only

Supplemental Table 5

Diagnostic results including RMSE, residual plots, ACF, and PACF plots

	Model 1	Model 2	Model 3
In-sample RMSE			
	0.165	0.073	0.073

Residual plots

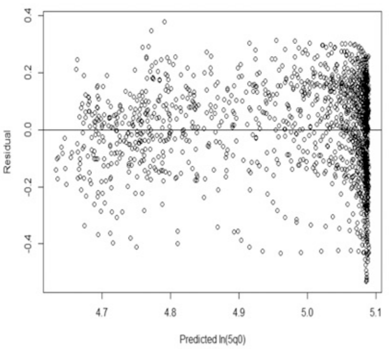

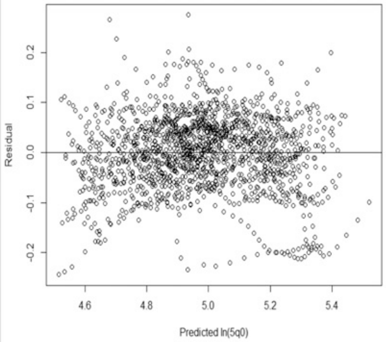

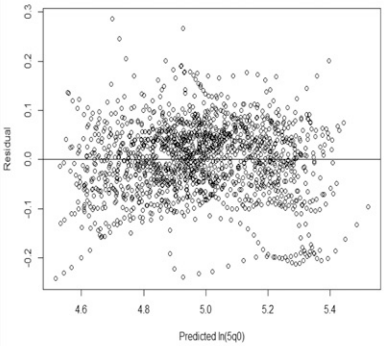

Autocorrelation function (ACF) plots

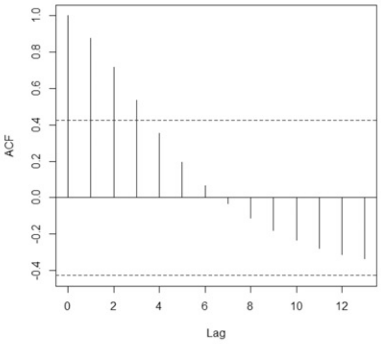

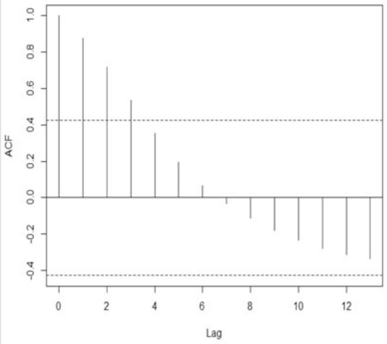

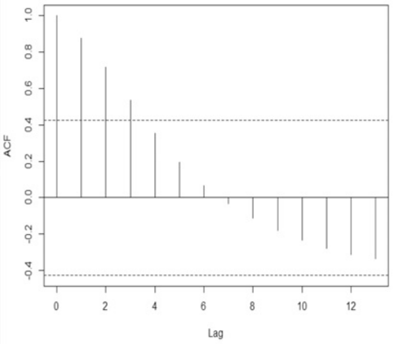

Partial autocorrelation function (PACF) plots

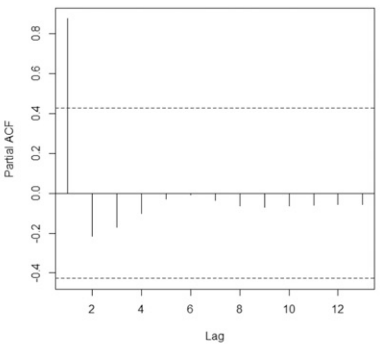

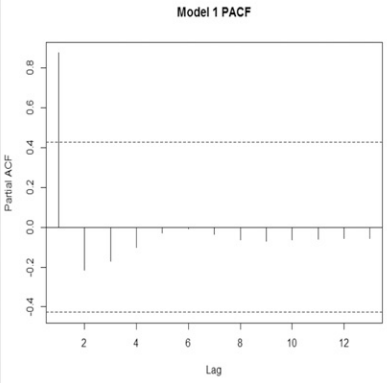

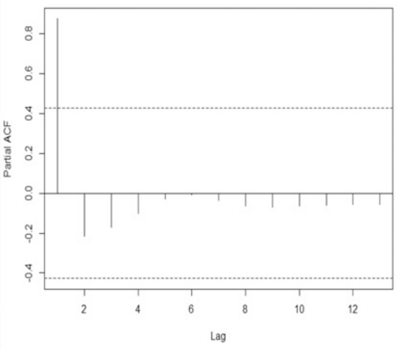

RMSE = root mean squared error.

random, indicating a relatively better fit to the data. Autocorrelation and partial autocorrelation function (ACF and PACF) plots showed that significant serial correlation existed in the residuals, providing justification for the use of bootstrapping in making statistical inference.

Cross-validation. One concern with fitting complex regression models was the potential of overfitting, which can lead to poor out-of-sample prediction. To address this issue, cross-validation analyses took place through which 20% of the 72 districts (i.e., 14 districts) were randomly held out as a test set and

The following are supplemental materials and will be published online only

Supplemental Table 6

Out-of-sample RMSE from cross-validation

Indicator	Model 1	Model 2	Model 3
Out-of-sample RMSE	0.1681	0.0839	0.0839

RMSE = root mean squared error.

the remaining districts were used as a training set. Each model was fitted to the training set to obtain coefficient estimates, after which the estimated equation was also applied to the test set and out-of-sample RMSE was calculated (Supplemental Table 6). As the results indicate, Models 2 and 3 offered considerably better out-of-sample prediction than Model 1.

Sensitivity analyses. We conducted sensitivity analyses to further validate the robustness of the final model (Model 3). Specifically, we subset the data in two ways to test model sensitivity to data perturbation. First, we subset the data with respect to time by restricting the time range between 2000 and 2010. Second, we subset the district data by categories of malaria transmission intensity.[2] For the latter, we subset transmission data by district boundaries and classified them accordingly: low ($PfPR_{2-10} \leq 5\%$), medium ($PfPR_{2-10} > 5\%$ and $< 40\%$), and high ($PfPR_{2-10} \geq 40\%$) transmission intensity.

Supplemental Table 7

Out-of-sample RMSE from sensitivity analyses

	Scenario 1	Scenario 2
Indicator	Data from 2000 to 2010	Medium-high vs. low transmission
Out-of-sample RMSE	0.0868	Medium-high: 0.0870<br>Low: 0.0854

RMSE = root mean squared error.

Supplemental Table 8

Regression coefficients by transmission intensity

	Low transmission ($PfPR_{2-10} \leq 5\%$)	Medium–high transmission ($PfPR_{2-10} > 5\%$)
Indicator	Beta (SE)	Beta (SE)
(Intercept)	31.109 (5.995)*	35.932 (3.726)*
SES	–0.218 (0.113)	–0.114 (0.101)
Composite of rapidly scaled up interventions	–0.029 (0.008)*	–0.025 (0.007)*
ANC1	–0.122 (0.099)	–0.119 (0.086)
DPT3	–0.145 (0.096)	–0.173 (0.044)*
Measles	0.074 (0.179)	0.162 (0.097)
Not underweight	–0.070 (0.166)	–0.259 (0.185)
Year	–0.013 (0.003)*	–0.015 (0.002)
Composite of rapidly scaled up interventions: SES	–0.002 (0.003)	0.004 (0.002)
Not underweight: SES	0.223 (0.155)	0.110 (0.119)

ANC1 = antenatal care, one visit; DPT3 = diphtheria-pertussis-tetanus vaccine, three doses; SES = socioeconomic status.
*$P < 0.05$.

High transmission districts were then combined with medium transmission districts. The out-of-sample RMSE for each scenario (Supplemental Table 7) did not substantially differ from the RMSE calculated for Model 3 during cross-validation analyses. This result shows that Model 3 was adequately resistant to data perturbation.

Section V. Other model specifications. In addition to the three models presented above, other specifications were also explored. Supplemental Table 9 summarizes some of the models considered and corresponding in-sample RMSEs.

Supplemental Table 9
RMSE for alternative models

	Model	RMSE
1-year lagged measles variable	$\ln(5q0)_{i,k,t} = \beta_0 + \beta_1\text{Scaled}_{i,t} + \beta_2\text{SES}_{i,t} + \beta_3\text{Und}_{i,t} + \beta_4\text{Scaled}_{i,t} \times \text{SES}_{i,t} + \beta_5\text{Und}_{i,t} \times \text{SES}_{i,t} + \beta_6\text{ANC1}_{i,t} + \beta_7\text{DPT3}_{i,t} + \beta_8\text{Meas}_{i,t-1} + \beta_9 t + \mu_k + \varepsilon_{i,k,t}$	0.0839
Year fixed effects	$\ln(5q0)_{i,k,t} = \beta_0 + \beta_1\text{Scaled}_{i,t} + \beta_2\text{SES}_{i,t} + \beta_3\text{Und}_{i,t} + \beta_4\text{Scaled}_{i,t} \times \text{SES}_{i,t} + \beta_5\text{Und}_{i,t} \times \text{SES}_{i,t} + \beta_6\text{ANC1}_{i,t} + \beta_7\text{DPT3}_{i,t} + \beta_8\text{Meas}_{i,t} + \beta_9 t + \gamma_k + \varepsilon_{i,k,t}$ where γ_k are province random effects.	0.0845
3-year period dummies	$\ln(5q0)_{i,k,t} = \beta_0 + \beta_1\text{Scaled}_{i,t} + \beta_2\text{SES}_{i,t} + \beta_3\text{Und}_{i,t} + \beta_4\text{Scaled}_{i,t} \times \text{SES}_{i,t} + \beta_5\text{Und}_{i,t} \times \text{SES}_{i,t} + \beta_6\text{ANC1}_{i,t} + \beta_7\text{DPT3}_{i,t} + \beta_8\text{Meas}_{i,t} + \sum_{j=9}^{17} \beta_j I_j + \gamma_k + \varepsilon_{i,k,t}$ where I_j are dummy indicators demarcating every 3-year period.	0.0854
Without control for secular trends	$\ln(5q0)_{i,k,t} = \beta_0 + \beta_1\text{Scaled}_{i,t} + \beta_2\text{SES}_{i,t} + \beta_3\text{Und}_{i,t} + \beta_4\text{Scaled}_{i,t} \times \text{SES}_{i,t} + \beta_5\text{Und}_{i,t} \times \text{SES}_{i,t} + \beta_6\text{ANC1}_{i,t} + \beta_7\text{DPT3}_{i,t} + \beta_8\text{Meas}_{i,t} + \gamma_k + \varepsilon_{i,k,t}$	0.0929
First differences	$\ln(5q0)_{i,k,t} - \ln(5q0)_{i,k,t-1} = \beta_0 + \beta_1\text{Scaled}_{i,t} + \beta_2\text{SES}_{i,t} + \beta_3\text{Und}_{i,t} + \beta_4\text{Scaled}_{i,t} \times \text{SES}_{i,t} + \beta_5\text{Und}_{i,t} \times \text{SES}_{i,t} + \beta_6\text{ANC1}_{i,t} + \beta_7\text{DPT3}_{i,t} + \beta_8\text{Meas}_{i,t} + \gamma_k + \varepsilon_{i,k,t}$	0.091
4-knot spline to control for secular trends	$\ln(5q0)_{i,k,t} = \beta_0 + \beta_1\text{Scaled}_{i,t} + \beta_2\text{SES}_{i,t} + \beta_3\text{Und}_{i,t} + \beta_4\text{Scaled}_{i,t} \times \text{SES}_{i,t} + \beta_5\text{Und}_{i,t} \times \text{SES}_{i,t} + \beta_6\text{ANC1}_{i,t} + \beta_7\text{DPT3}_{i,t} + \beta_8\text{Meas}_{i,t} + \beta_9 B_{t1} + \beta_{10} B_{t2} + \beta_{11} B_{t3} + \beta_{12} B_{t4} + \gamma_k + \varepsilon_{i,k,t}$ where B_{t1}–B_{t4} are the spline basis.	0.0876

RMSE = root mean squared error.

SUPPLEMENTAL REFERENCES

1. Hall P, 1986. On the number of bootstrap simulations required to construct a confidence interval. *Ann Stat 14:* 1453–1462.
2. Gething PW, Patil AP, Smith DL, Guerra CA, Elyazar IR, Johnston GL, Tatem AJ, Hay SI, 2011. A new world malaria map: *Plasmodium falciparum* endemicity in 2010. *Malar J 10:* 378.
